# Supplementary material for: Mechanism of LolCDE as a molecular extruder of bacterial triacylated lipoproteins
Source: Nat Commun. 2021 Aug 3;12:4687. doi: 10.1038/s41467-021-24965-1 (PMC8333309; doi:10.1038/s41467-021-24965-1)
Supplement: Supplementary file 1 — Supplementary Information [file 41467_2021_24965_MOESM1_ESM.pdf]

Mechanism of LolCDE as a molecular extruder of bacterial triacylated lipoproteins

Stuti Sharma[1,*], Ruoyu Zhou[2,*], Li Wan[2], Shan Feng[2], KangKang Song[3,4], Chen Xu[3,4], Yanyan Li[2,#] & Maofu Liao[1,#]

[1]Department of Cell Biology, Blavatnik Institute, Harvard Medical School, Boston MA, USA
[2]Key Laboratory of Structural Biology of Zhejiang Province, School of Life Sciences, Westlake University, Hangzhou 310024, China
[3]Department of Biochemistry and Molecular Pharmacology, University of Massachusetts Medical School, Worcester MA, USA
[4]Cryo-EM Core Facility, University of Massachusetts Medical School, Worcester MA, USA

*These authors contributed equally to this work

[#]Correspondence to: liyanyan@westlake.edu.cn & maofu_liao@hms.harvard.edu

Supplementary Information:

Supplementary Figures 1-9

Supplementary Tables 1-2

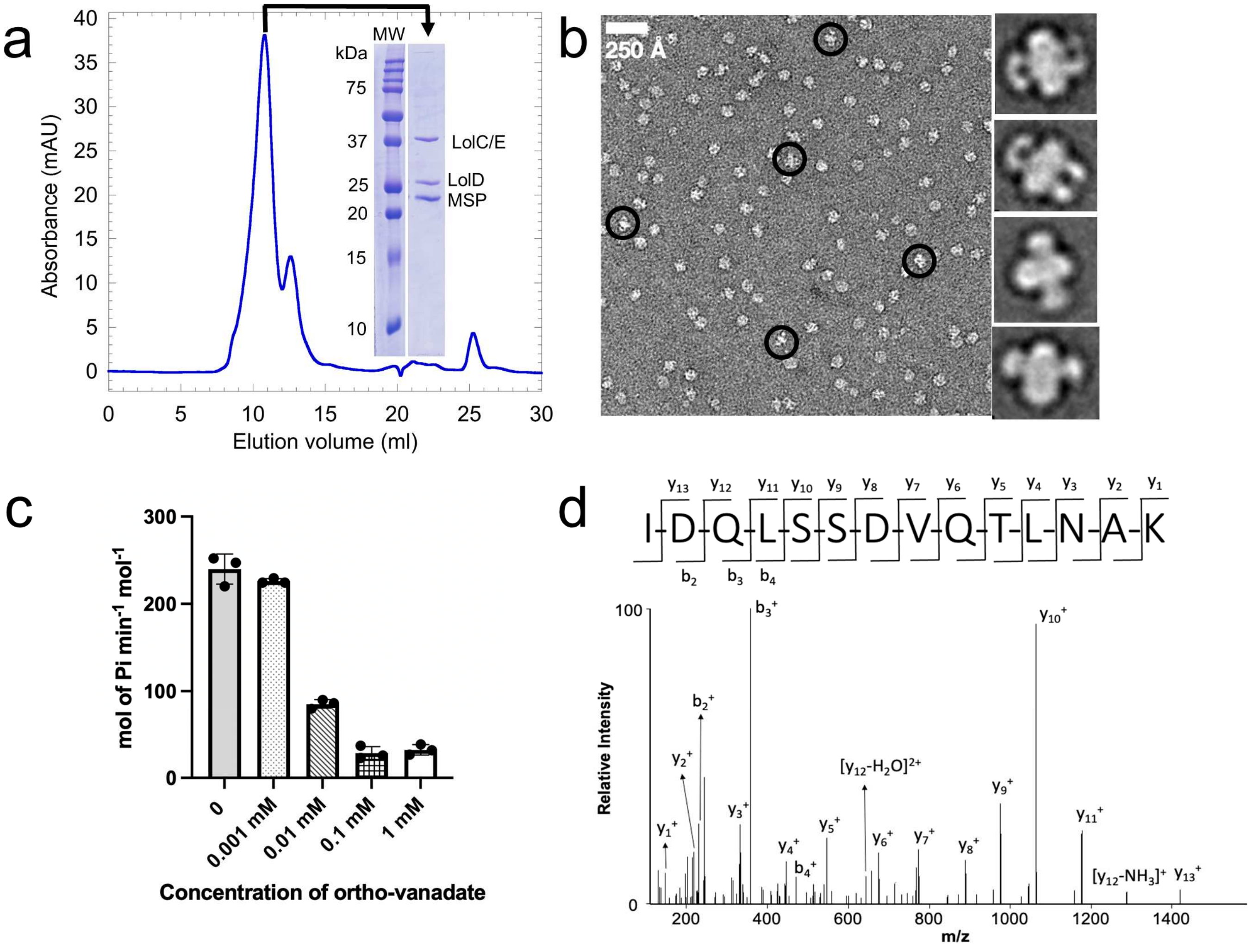

Supplementary Figure 1. Purification and functional characterization of LolCDE in nanodiscs. a, Size exclusion chromatography profile of LolCDE in lipid nanodiscs and SDS-PAGE of the peak fraction shown in inset. This experiment was repeated three times independently with similar results. **b**, Representative negative-stain EM image and 2D class averages of LolCDE in nanodiscs. This experiment was repeated three times independently with similar results. **c**, Inhibition of ATPase activity of nanodisc-embedded LolCDE with increasing concentration of sodium orthovanadate. Each data point is the average of three measurements, and error bars represent the s.d. **d**, Mass spectrometry identification of Lpp peptide. Shown is the MS/MS spectrum of duple charged IDQLSSDVQTLNAK peptide ion, which represents one of the unique peptides in protein Lpp. The labeled peaks correspond to masses of y ions and b ions of the selected peptide. Mass spectrometry raw data are available as Source Data.

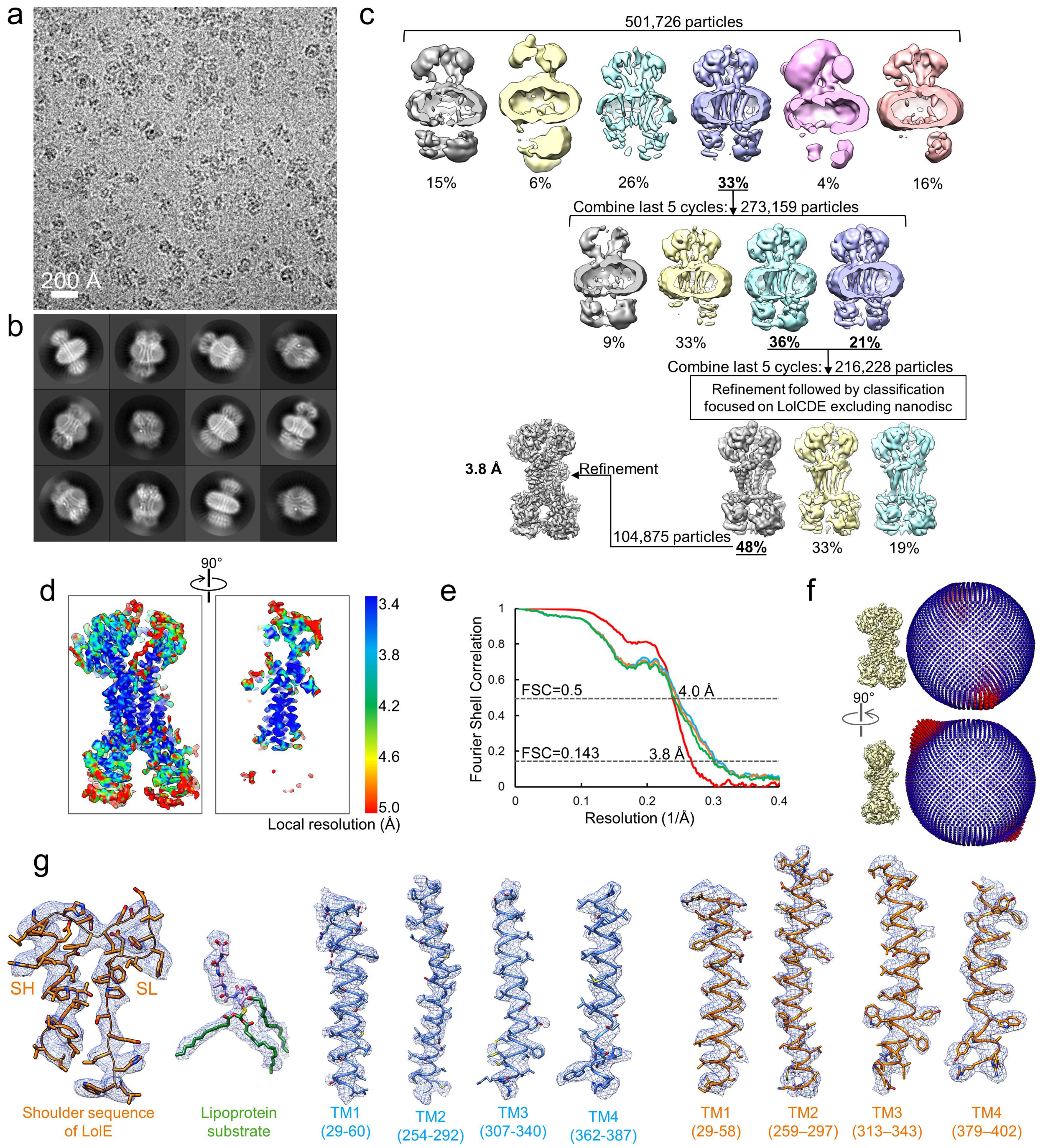

Supplementary Figure 2. Single-particle cryo-EM study of nucleotide-free LolCDE in nanodiscs. **a**, Representative cryo-EM image of LolCDE in nanodiscs. This experiment was repeated two times independently with similar results. **b**, Two-dimensional class averages of cryo-EM particle images. **c**, Three-dimensional classification and refinement of cryo-EM particle images. **d**, Local resolution of the final cryo-EM map. **e**, Fourier shell correlation (FSC) curves: gold-standard FSC curve between the two half maps with indicated resolution at FSC = 0.143 (red); FSC curve between the model and the final map with indicated resolution at FSC = 0.5 (blue); FSC curve between half map 1 (orange) or half map 2 (green) and the model refined against half map 1. **f**, Angular distribution of particle images included in the final 3D reconstruction. **g**, Superimposition of cryo-EM densities and the model for selected regions.

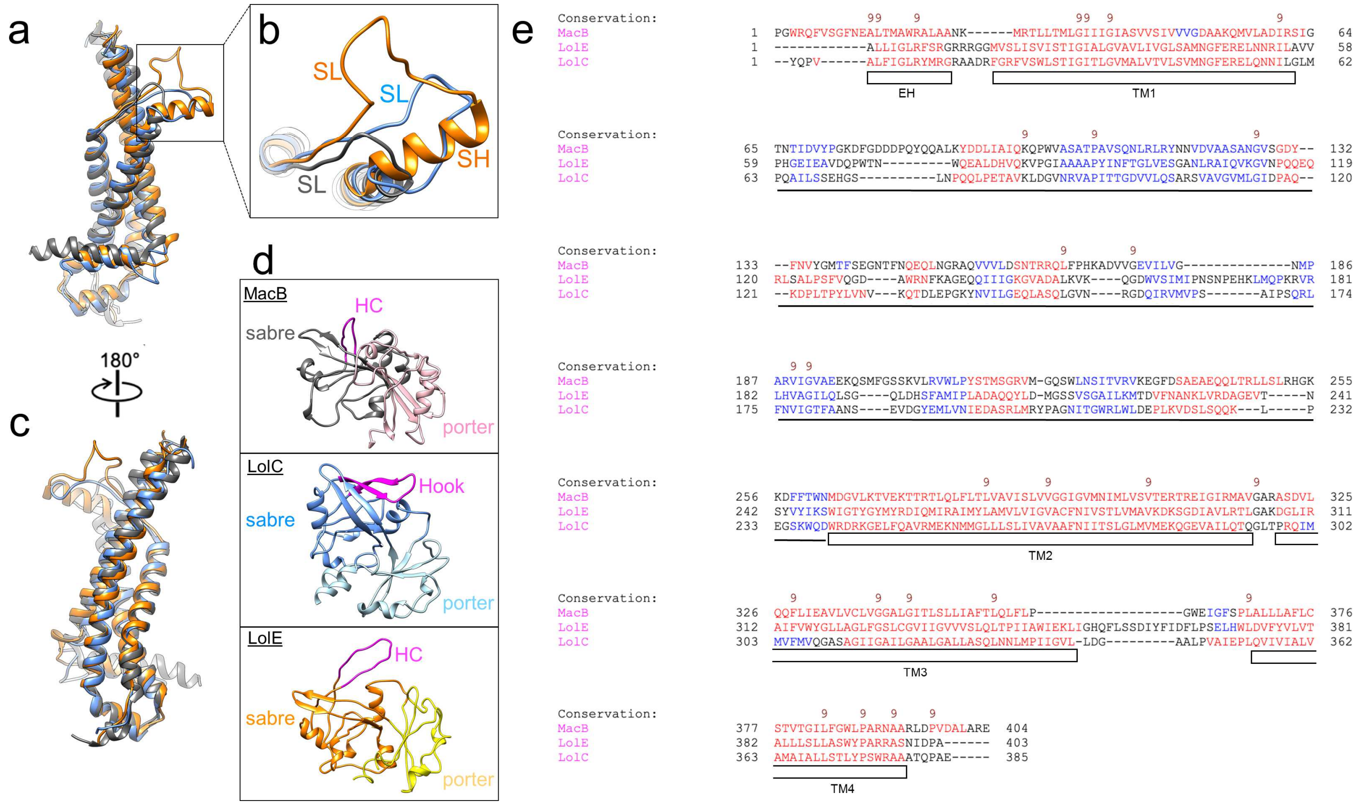

Supplementary Figure 3. Comparison of LolC, LolE and MacB. a, Superimposition of the TMDs of LolC (blue), LolE (orange) and MacB (grey, PDB: 5LIL). **b**, Close-up view of the shoulder loop (SH) region. **c**, 180° rotated view of (**a**). **d**, Structures of the periplasmic domains of MacB, LolC and LolE, oriented based on TMD alignment in (**a**). The porter and sabre subdomains, as well as Hook (in LolC) and Hook counterparts (HC, in MacB and LolE) are highlighted. **e**, Sequence alignment of LolC, LolE and MacB from *E. coli*, conducted using PROMALS3D. Predicated secondary structure elements are colored in red (α-helix) and blue (β-strand). The first line in each block shows conservation indices for positions with a conservation index above 4 (9 being highly conserved).

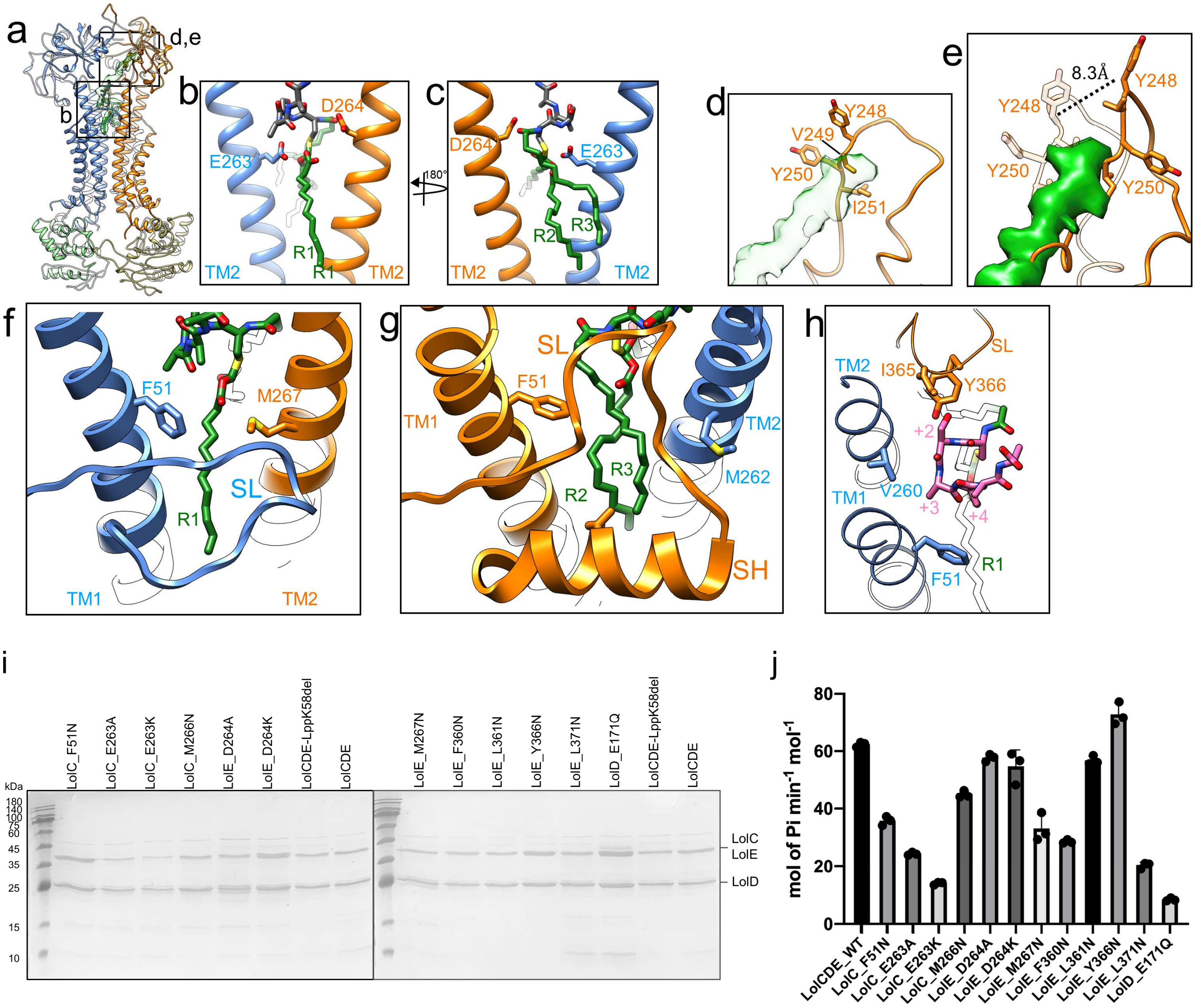

Supplementary Figure 4. Structural details of LolCDE. **a**, Structure of nucleotide-free LolCDE in nanodiscs. **b**, **c**, Close-up views of lipoprotein binding pockets as indicated in (**a**), showing two negatively charged residues near +1 cysteine. **d**, Close-up view of the lipoprotein linker interacting loop in the periplasmic domain of LolE, as indicated in (**a**), with the linker in transparent green. **e**, Same region as in (**d**), shown as superimposition of nucleotide-free (transparent) and vanadate-trapped (solid) conformation. The displacement of Tyr248 upon vanadate trapping is indicated. **f**, Lipoprotein binding front pocket enclosing acyl chain R1. The distance between the Cα atoms of LolC-F51 and LolE-M267 is 14.9 Å. **g**, Lipoprotein binding back pocket enclosing acyl chains R2 and R3. The distance between LolE-F51 and LolC-M262 is 17.6 Å. **h**, N-terminal lipoprotein peptide enclosing pocket composed of LolC-TM1, LolC-TM2 and LolE-TM2. **i**, SDS-PAGE of purified LolCDE and single-residue mutants in DDM. **j**, ATPase activity of purified LolCDE and mutants in DDM. Shown is a representative of three independent experiments. Each point represents mean ± s.d. of three measurements in one experiment.

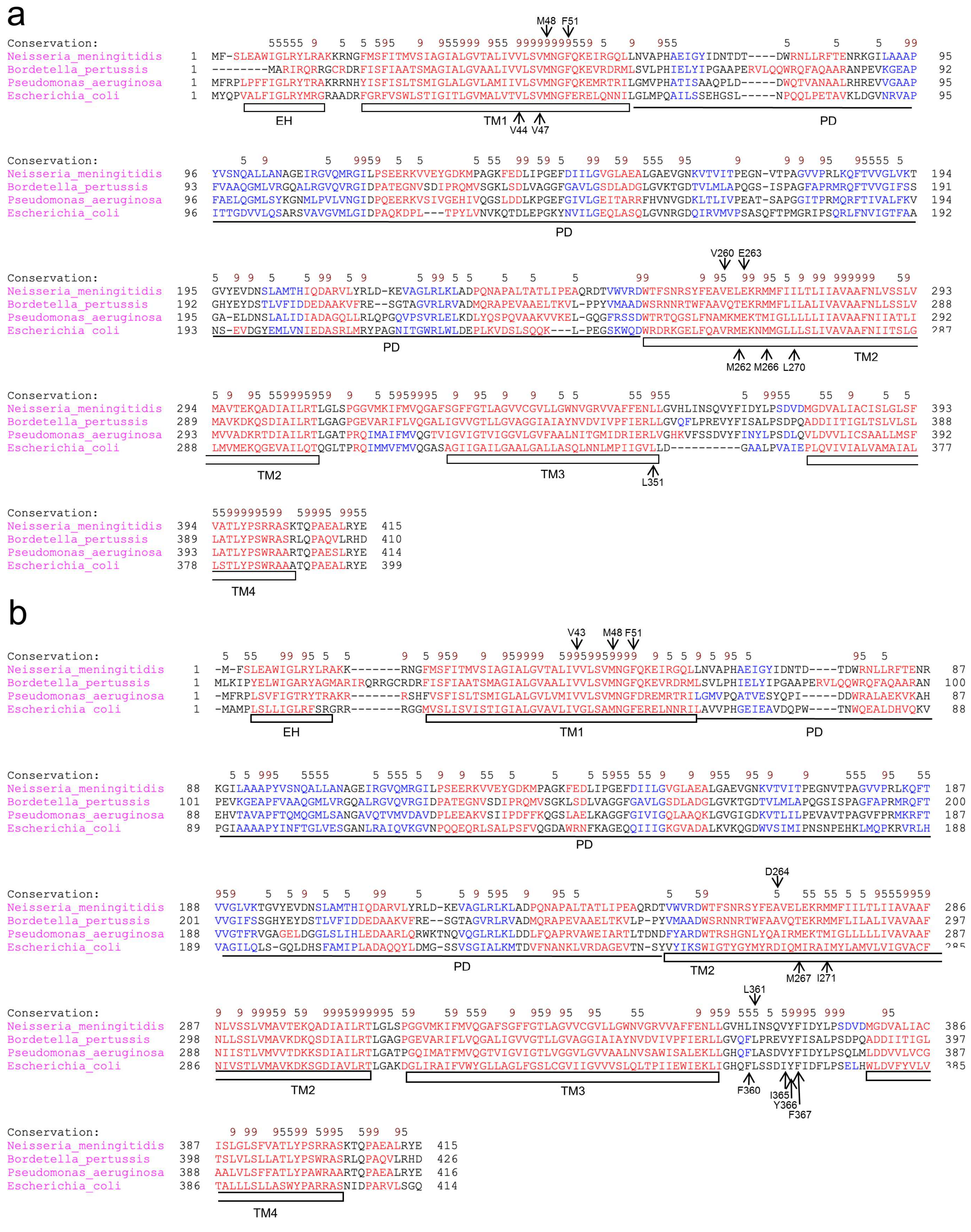

Supplementary Figure 5. Multiple sequence alignment for LolC and LolE. a, Alignment of the amino acid sequences of LolC from four different Gram-negative bacteria. The amino acid residues involved in lipoprotein binding are indicated. The alignment was conducted using PROMALS3D. Predicated secondary structure elements are colored in red (α-helix) and blue (β-strand). **b**, Same as (**a**), except for LolE. The first line in each block shows conservation indices for positions with a conservation index above 4 (9 being highly conserved).

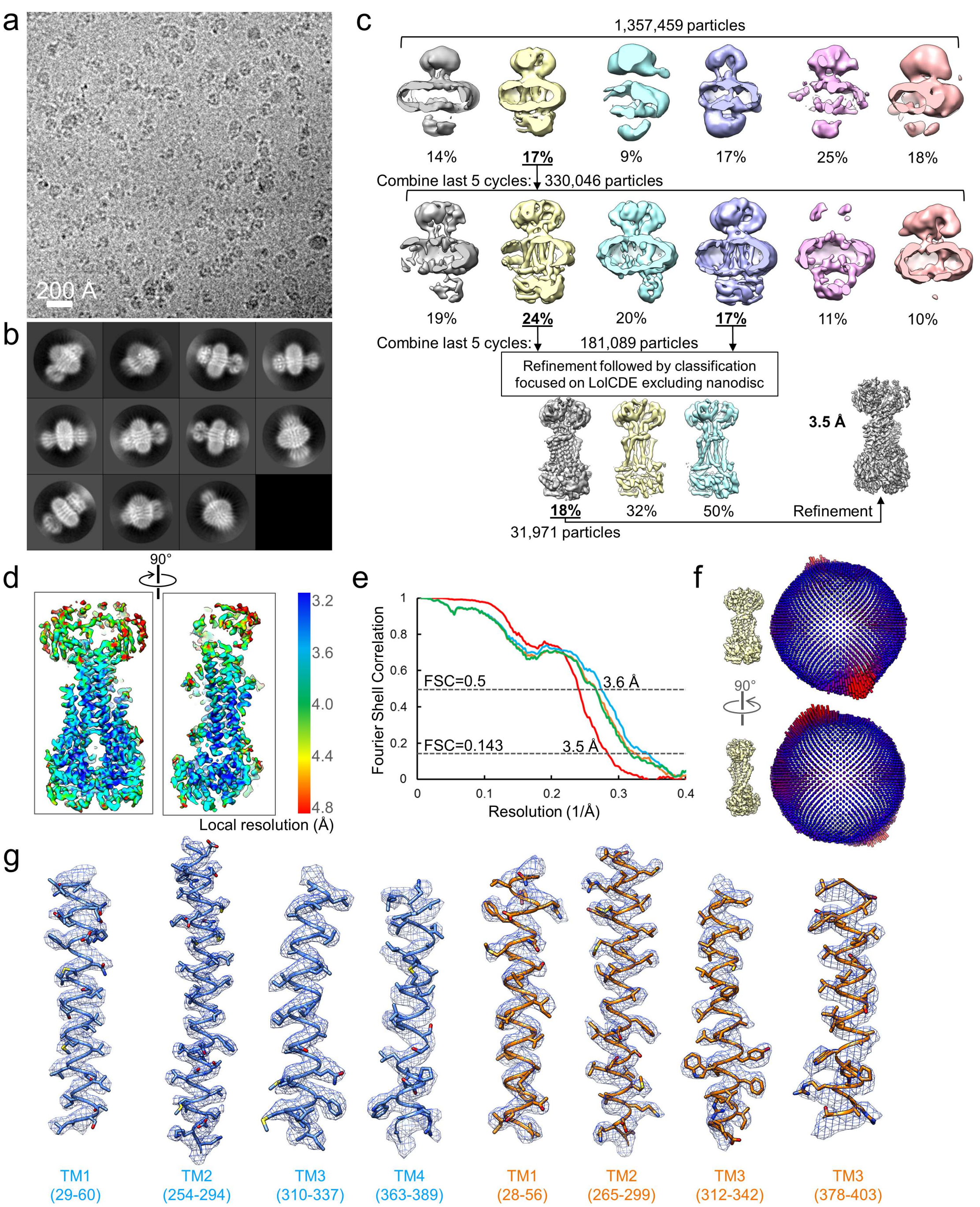

Supplementary Figure 6. Single-particle cryo-EM study of vanadate-trapped LolCDE in nanodiscs. a, Representative cryo-EM image of nanodisc-embedded LolCDE with vanadate trapping. This experiment was repeated two times independently with similar results. **b**, Two-dimensional class averages of cryo-EM particle images. **c**, Three-dimensional classification and refinement of cryo-EM particle images. **d**, Local resolution of the final cryo-EM map. **e**, Fourier shell correlation (FSC) curves: gold-standard FSC curve between the two half maps with indicated resolution at FSC = 0.143 (red); FSC curve between the model and the final map with indicated resolution at FSC = 0.5 (blue); FSC curve between half map 1 (orange) or half map 2 (green) and the model refined against half map 1. **f**, Angular distribution of particle images included in the final 3D reconstruction. **g**, Superimposition of cryo-EM densities and the model for selected regions.

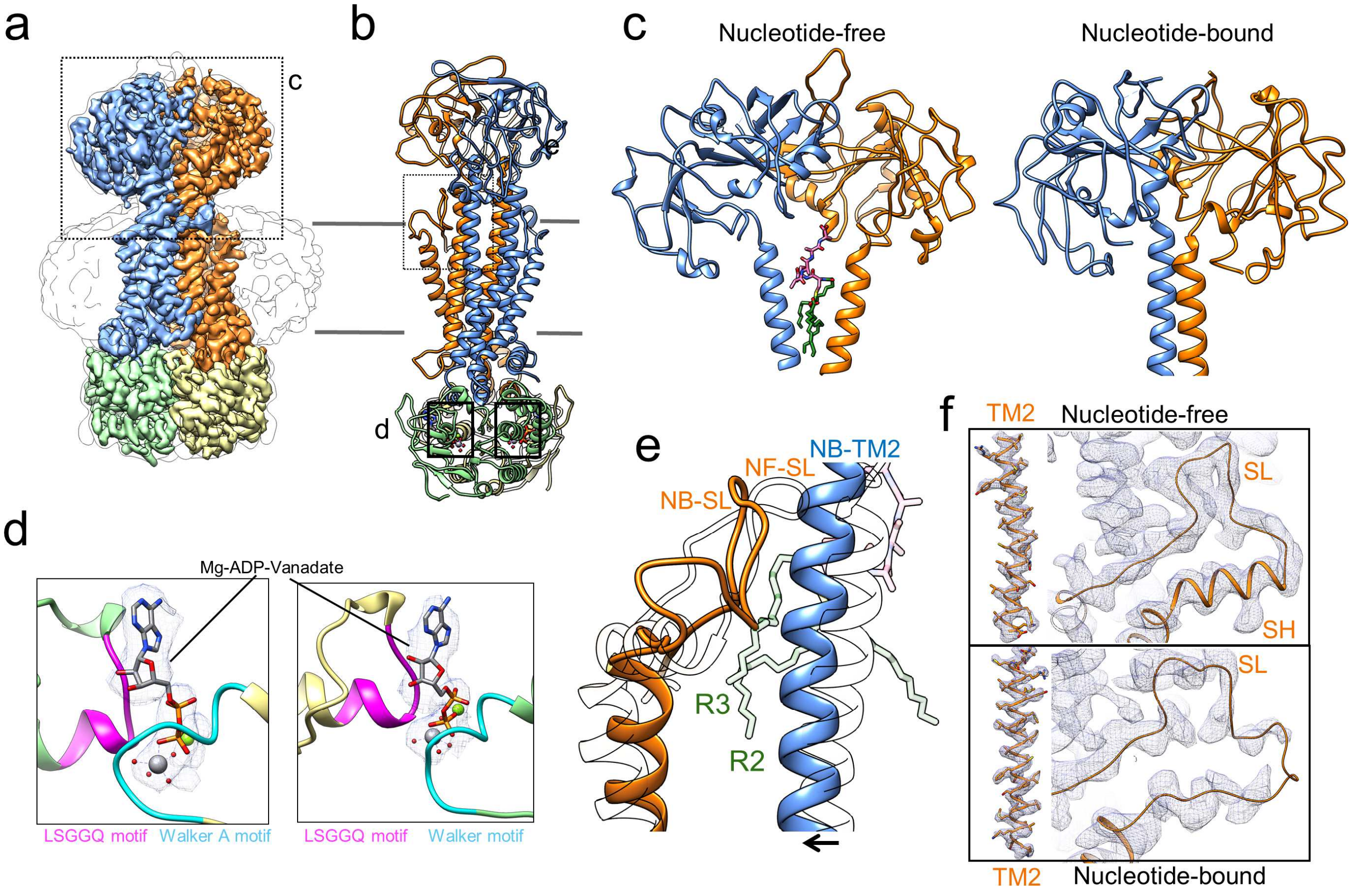

Supplementary Figure 7. Conformational changes of LolCDE induced by vanadate trapping. a, Surface side view of cryo-EM map of vanadate-trapped LolCDE in nanodiscs, filtered at 3.5-Å resolution and colored as in Fig. 1. **b**, 90° rotated side view of model. **c**, Periplasmic region of LolCDE as indicated in (**a**), in nucleotide-free and vanadate-trapped conformations. **d**, Close-up views of the model of two ATP binding sites as indicated in (**b**), shown as superimposition with the EM density of ADP-vanadate complex that is trapped between Walker A and signature motif. **e**, Comparison of the nucleotide-free and vanadate-trapped LolCDE in the region of the shoulder loop (SL) of LolE, as indicated in (**b**), showing blockage of substrate access to SL by the inward movement of the TM2 of LolC. Nucleotide-free conformation is shown as transparent. **f**, Cryo-EM density (gray mesh, contoured at 5.4σ) of the shoulder helix (SH) and SL of LolE in the nucleotide-free (top) and vanadate-trapped (bottom) conformations. The EM densities of the TM2 of LolE in two conformations are contoured at 5.4σ for comparison.

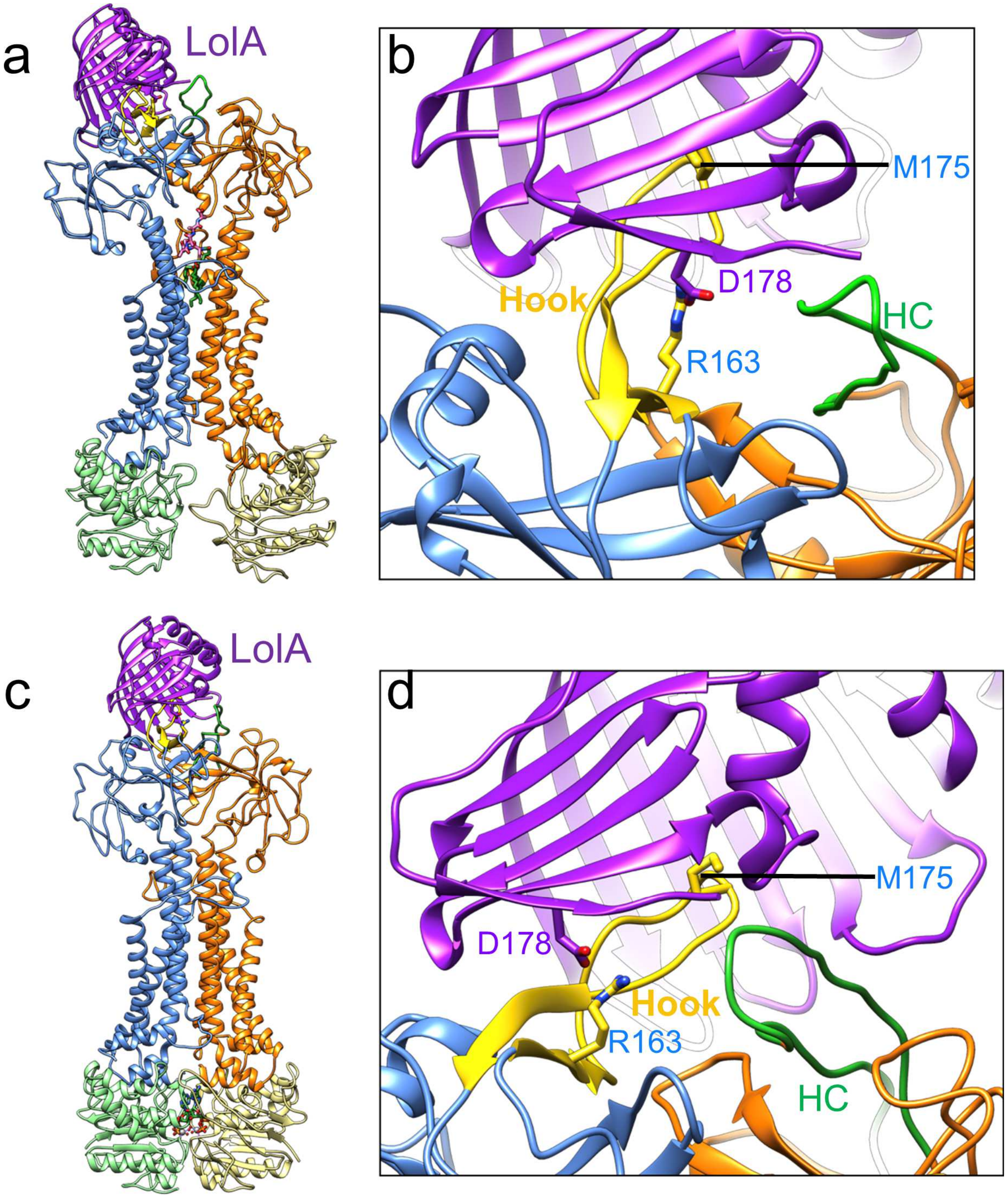

Supplementary Figure 8. Composite models of LolCDE-LolA complex. **a**, Composite model of LolCDE-LolA in nucleotide-free state, generated by docking LolA on the nucleotide-free conformation of LolCDE. The periplasmic domain of LolC (LolC-PD) from the crystal structure of the LolC-PD-LolA complex (PDB: 6F3Z) was superimposed with LolC-PD in LolCDE. **b**, Close-up view of the binding interface of LolC-LolA with the Hook and pad on LolC highlighted in yellow. The Hook conterpart in LolE is colored green. Key residues implicated in LolA-LolC interaction are indicated. **c, d**, Same as (**a**) and (**b**), except for vanadate-trapped conformation of LolCDE.

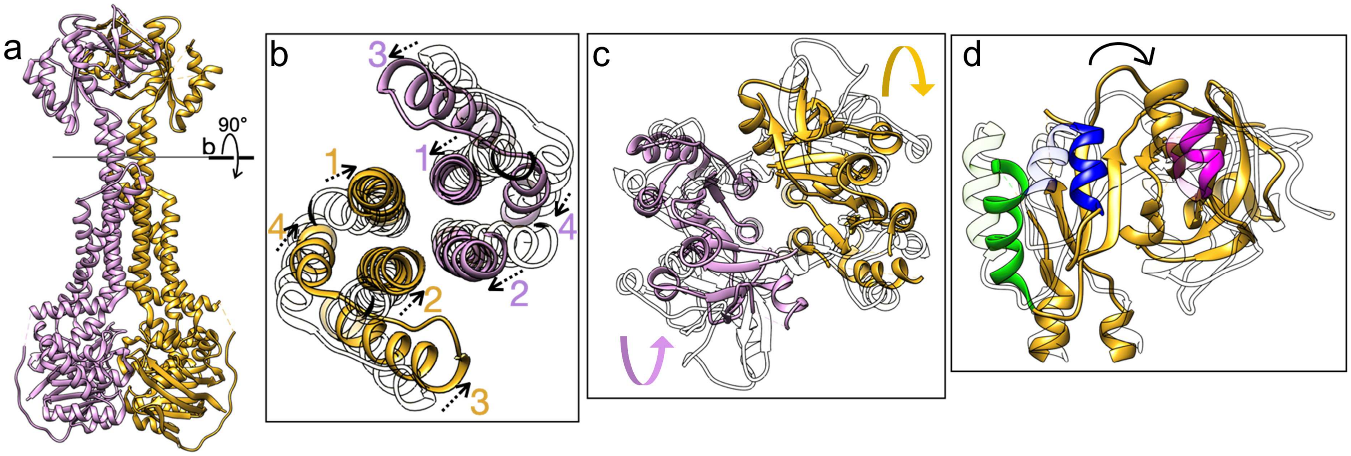

Supplementary Figure 9. Conformational changes of MacB induced by ATP binding. a, MacB in ATP-bound conformation (PDB: 5LJ7). **b**, Sectional view showing TM helices in the nucleotide-free (transparent) and nucleotide-bound (color) conformations. TM2 undergoes an inward and upward conformational shift, while TM1, TM3 and TM4 all shift inward. **c**, Top-down view showing the motion of the periplasmic domains in nucleotide-free (transparent) and nucleotide-bound (color) conformation. The direction of rotation is indicated by arrows. **d**, Side view of one periplasmic domain, highlighting the shifts of various structural elements.

Supplementary Table 1. Cryo-EM structure determination parameters and model statistics.

	Nucleotide-free LolCDE	Vanadate-trapped LolCDE
	PDB ID: 7MDX	PDB ID: 7MDY
	EMDB ID: 23783	EMDB ID: 23784

Data collection and processing

Microscope	Titan Krios (UMMS)	Titan Krios (Westlake)
Detector	K3 Summit	K3 Summit
Magnification	81,000	81,000
Voltage (kV)	300	300
Electron exposure ($e^-/Å^2$)	46.4	50
Defocus range (μm)	-1 to -2.5	-1 to -2.2
Pixel size (Å)	1.06	1.087
Symmetry imposed	C1	C1
Initial particle images (no.)	501,726	1,357,459
Final particle images (no.)	104,875	31,971
Map resolution (Å)	3.8	3.5
FSC threshold	0.143	0.143
Map resolution range (Å)	3.4 - 5.0	3.2 - 4.8

Refinement

Initial model used (pdb id)	5LIL, 6F3Z	5LIL, 6F3z
Model resolution (Å)<br>FSC threshold	4.0<br>0.5	3.6<br>0.5
Map sharpening B factor (Å)	N/A	-126
Model composition<br>Non-hydrogen atoms<br>Protein residues<br>Ligands<br>Mg	 9507<br>1236<br>1<br>0	 9741<br>1266<br>2<br>2
B-factors (Å)<br>Protein<br>Ligand	 150.4<br>104.58	 60.96<br>38.28
R.M.S deviations<br>Bond length (Å)<br>Bond angles (°)	 0.002<br>0.549	 0.004<br>0.772
Validation<br>MolProbity score<br>Clashscore<br>Poor rotamers (%)	 2.08<br>11.19<br>0.00	 2.37<br>9.38<br>2.41
Ramachandran plot<br>Favored (%)<br>Allowed (%)<br>Outliers (%)	 91.01<br>8.99<br>0.00	 88.75<br>11.17<br>0.08

Supplementary Table 2. Primers used in this study. Shown below are the primers used for site-directed mutagenesis. Forward and reverse primers are listed for each individual point mutant.

Primer	Sequence
LolC_F51N_Forward	5'-AGCGCGAGCTGCAAAA-3'
LolC_F51N_Reverse	5'-CATTGCCGTTCATCACTGACAAT-3'
LolC_E263A_Forward	5'-GCAAAAAATATGATGGGTTTACTGCTG-3'
LolC_E263A_Reverse	5'-CATGCGTACGGCCTG-3'
LolC_E263K_Forward	5'-AAAAAAAATATGATGGGTTTACTGCT-3'
LolC_E263K_Reverse	5'-CATGCGTACGGCCTG-3'
LolC_M266N_Forward	5'-AATATGGGTTTACTGCTGAGC-3'
LolC_M266N_Reverse	5'-ATTTTTTTCCATGCGTACGG-3'
LolE_D264A_Forward	5'-GCTATCCAAATGATCCGCGCC-3'
LolE_D264A_Reverse	5'-GCGATACATATAGCCGTAAGTAC-3'
LolE_D264K_Forward	5'-AAAATCCAAATGATCCGCGCC-3'
LolE_D264K_Reverse	5'-GCGATACATATAGCCGTAAGTAC-3'
LolE_M267N_Forward	5'-AACATCCGCGCCATTATGTATC-3'
LolE_M267N_Reverse	5'-TTGGATATCGCGATACATATAGCC-3'
LolE_F360N_Forward	5'-CTCTCCAGCGATATCTATTTTATTGAT-3'
LolE_F360N_Reverse	5'-GTTCTGATGACCGATCAACTTTTCAA-3'
LolE_L361N_Forward	5'-TCCAGCGATATCTATTTTATTGATTTCC-3'
LolE_L361N_Reverse	5'-GTTGAACTGATGACCGATCAACT-3'
LolE_Y366N_Forward	5'-TTTATTGATTTCCTGCCATCGG-3'
LolE_Y366N_Reverse	5'-ATTGATATCGCTGGAGAGGAAC-3'
LolE_L371N_Forward	5'-AACCCATCGGAATTGCACTGG-3'
LolE_L371N_Reverse	5'-GAAATCAATAAAATAGATATCGCTGGAGAG-3'
LolD_E171Q-Forward	5'-CAACCTACCGGTAACCTCG-3'
LolD_E171Q-Reverse	5'-ATCCGCCAGTACCAGG-3'
