# Supplementary material for: Mechanism of LolCDE as a molecular extruder of bacterial triacylated lipoproteins
Source: Nat Commun. 2021 Aug 3;12:4687. doi: 10.1038/s41467-021-24965-1 (PMC8333309; doi:10.1038/s41467-021-24965-1)
Supplement: Supplementary file 3 — Description of Additional Supplementary Files [file 41467_2021_24965_MOESM3_ESM.pdf]

Description of Additional Supplementary Files

File Name: Supplementary Movie 1
Description: **Conformational transition between nucleotidefree and vanadate-trapped E. coli LolCDE in nanodiscs.** The animation shows a morph between the nucleotide-free and nucleotide-bound states of LolCDE. The subunits are color coded as in the manuscript figures with the LolC-hook shown in magenta. The lipoprotein substrate and ADP-vanadate are omitted.

File Name: Supplementary Movie 2
Description: **Conformational transition between nucleotidefree and ATP-bound MacB.** The animation shows a morph between the nucleotide-free (PDB: 5NIL) and ATP-bound (PDB: 5LJ7) conformations of MacB.
