# Supplementary material for: Mechanism of LolCDE as a molecular extruder of bacterial triacylated lipoproteins
Source: Nat Commun. 2021 Aug 3;12:4687. doi: 10.1038/s41467-021-24965-1 (PMC8333309; doi:10.1038/s41467-021-24965-1)
Supplement: Supplementary file 6 — Reporting Summary [file 41467_2021_24965_MOESM6_ESM.pdf]

nature portfolio

Corresponding author(s): Yanyan Li, Maofu Liao

Last updated by author(s): Jul 13, 2021

Reporting Summary

Nature Portfolio wishes to improve the reproducibility of the work that we publish. This form provides structure for consistency and transparency in reporting. For further information on Nature Portfolio policies, see our Editorial Policies and the Editorial Policy Checklist.

Statistics

For all statistical analyses, confirm that the following items are present in the figure legend, table legend, main text, or Methods section.

n/a	Confirmed	
☐	☒	The exact sample size (*n*) for each experimental group/condition, given as a discrete number and unit of measurement
☐	☒	A statement on whether measurements were taken from distinct samples or whether the same sample was measured repeatedly
☒	☐	The statistical test(s) used AND whether they are one- or two-sided<br>*Only common tests should be described solely by name; describe more complex techniques in the Methods section.*
☒	☐	A description of all covariates tested
☒	☐	A description of any assumptions or corrections, such as tests of normality and adjustment for multiple comparisons
☐	☒	A full description of the statistical parameters including central tendency (e.g. means) or other basic estimates (e.g. regression coefficient) AND variation (e.g. standard deviation) or associated estimates of uncertainty (e.g. confidence intervals)
☒	☐	For null hypothesis testing, the test statistic (e.g. *F*, *t*, *r*) with confidence intervals, effect sizes, degrees of freedom and *P* value noted<br>*Give P values as exact values whenever suitable.*
☒	☐	For Bayesian analysis, information on the choice of priors and Markov chain Monte Carlo settings
☒	☐	For hierarchical and complex designs, identification of the appropriate level for tests and full reporting of outcomes
☒	☐	Estimates of effect sizes (e.g. Cohen's *d*, Pearson's *r*), indicating how they were calculated

Our web collection on statistics for biologists contains articles on many of the points above.

Software and code

Policy information about availability of computer code

Data collection	SerialEM (v3.7), AutoEMation (no version)
Data analysis	Phenix (v 1.16); Coot (v 0.8.9); MotionCor2 (v 1.1.0); CTFFIND4 (v 4.1.5); SAMUEL (v 21.01); SamViewer (v 21.01); SPIDER (v 17.05); RELION 3.0; ResMap (v 1.1.4); bfactor (v 1.03); Chimera (v 1.13); Graphpad Prism (v 9.1.0)

For manuscripts utilizing custom algorithms or software that are central to the research but not yet described in published literature, software must be made available to editors and reviewers. We strongly encourage code deposition in a community repository (e.g. GitHub). See the Nature Portfolio guidelines for submitting code & software for further information.

Data

Policy information about availability of data

All manuscripts must include a data availability statement. This statement should provide the following information, where applicable:

- Accession codes, unique identifiers, or web links for publicly available datasets
- A description of any restrictions on data availability
- For clinical datasets or third party data, please ensure that the statement adheres to our policy

The three-dimensional cryo-EM density maps of E. coli LolCDE in nanodiscs have been deposited in the Electron Microscopy Data Bank under accession numbers: EMD-23783 (nucleotide-free) and EMD-23784 (vanadate-trapped). Atomic coordinates for the atomic models of LolCDE have been deposited in the Protein Data Bank under accession numbers: 7MDX (nucleotide-free) and 7MDY (vanadate-trapped). Source data are provided with this paper.

Field-specific reporting

Please select the one below that is the best fit for your research. If you are not sure, read the appropriate sections before making your selection.

☒ Life sciences ☐ Behavioural & social sciences ☐ Ecological, evolutionary & environmental sciences

For a reference copy of the document with all sections, see nature.com/documents/nr-reporting-summary-flat.pdf

Life sciences study design

All studies must disclose on these points even when the disclosure is negative.

Sample size	No statistical methods were used to predetermine sample size. The sample size was determined based on sufficient EM data that can achieve adequate single-particle EM analysis and 3D cryo-EM reconstructions.
Data exclusions	No data were excluded from analyses.
Replication	Each experiment was repeated at least three times in independent experiments. Experimental findings were reproduced reliably.
Randomization	This is not relevant to our study, because no grouping was needed.
Blinding	Investigators were not blinded to group allocation, because no grouping was needed for this study.

Reporting for specific materials, systems and methods

We require information from authors about some types of materials, experimental systems and methods used in many studies. Here, indicate whether each material, system or method listed is relevant to your study. If you are not sure if a list item applies to your research, read the appropriate section before selecting a response.

Materials & experimental systems

n/a	Involved in the study	
☐	☒	Antibodies
☒	☐	Eukaryotic cell lines
☒	☐	Palaeontology and archaeology
☒	☐	Animals and other organisms
☒	☐	Human research participants
☒	☐	Clinical data
☒	☐	Dual use research of concern

Methods

n/a	Involved in the study	
☒	☐	ChIP-seq
☒	☐	Flow cytometry
☒	☐	MRI-based neuroimaging

Antibodies

Antibodies used	Anti-c-Myc tag mouse monoclonal antibody (CWBio, CW0299M, Lot 01217/20450, 1:2000)<br>Anti-His tag mouse monoclonal antibody (CWBio, CW0286M, Lot 01249/50449, 1:2000)<br>Goat anti-mouse IgG, HRP conjugated (CWBio, CW0102S, Lot 01325/10536, 1:10000)
Validation	Anti-c-Myc tag mouse monoclonal antibody was validated by the manufacturer (CWBio): the antibody detects a single band (~50 kDa) of a c-Myc tagged protein on a western blot from bacterial crude cell lysate.<br>Anti-His mouse monoclonal antibody was validated by the manufacturer (CWBio): the antibody specifically detects the proteins containing His tag on a western blot.<br>Goat anti-mouse IgG, HRP conjugated was validated by the manufacturer (CWBio): the antibody specifically detects mouse IgG heavy and light chains in ELISA, western blot, and immunohistochemistry.

March 2021
